# Numerical Simulation of Damage Processes in CCD Detectors Induced by Multi-Pulse Nanosecond Laser Irradiation

**DOI:** 10.3390/s25154851

**Published:** 2025-08-07

**Authors:** Weijing Zhou, Hao Chang, Zhilong Jian, Yingjie Ma, Xiaoyuan Quan, Chenyu Xiao

**Affiliations:** Department of Aerospace and Technology, Space Engineering University, Beijing 101416, China; viviazhouyy@163.com (W.Z.); jzl862366@163.com (Z.J.); mayingjie@hgd.edu.cn (Y.M.); quanxiaoyuan@hgd.edu.cn (X.Q.); 16605655985@163.com (C.X.)

**Keywords:** multi-pulse laser, nanosecond, laser irradiation, damage, numerical simulation, CCD detector

## Abstract

This paper presents a finite element simulation of thermal damage to a CCD caused by nanosecond multi-pulse laser exposure. The temperature changes in the CCD due to the laser pulses were simulated, and the time evolution of thermal damage was studied. The impacts of different laser parameters such as spot radius, pulse width, and repetition frequency on thermal damage were evaluated. The results indicated that the temperature of the CCD increased with each pulse due to cumulative effects, leading to thermal damage. A smaller laser spot size intensified the temperature rise, accelerating the rate at which different layers in the CCD exceeded the relative melting point of each material. In the case of nanosecond pulse width, variations in pulse width had minimal effects on CCD thermal damage when repetition frequency and average power density were constant. Lower repetition frequencies made it easier to cause melting damage to the CCD when pulse width and average power density were constant.

## 1. Introduction

A Charge Coupled Device (CCD) is known for its high resolution, sensitivity, and wide spectral coverage. The stability and reliability of its performance are crucial for camera imaging quality [1]. However, the CCD is highly susceptible to transient interference or permanent damage when exposed to laser irradiation. Here, interference refers to temporary degradation in detector performance that recovers over time, whereas damage denotes irreversible destruction of materials or structures. Consequently, mechanistic studies of laser–detector interactions remain critical for advancing optoelectronic countermeasures [2], laser dazzle protection [3,4], and reliability in industrial machine vision systems [5,6].

Researchers have conducted experiments and created physical models to investigate the damage mechanism of CCDs when irradiated by lasers. Some researchers have developed two-dimensional [7,8,9], two-dimensional axisymmetric [10,11], or three-dimensional finite element models [12,13] to study thermal damage in CCDs. Different types of laser sources, including single-pulse [14,15], multi-pulse [16], and continuous lasers [7,17], have also been studied for their effects on CCDs. The research has additionally assessed the damage threshold and examined the effects of parameters such as laser wavelength [18,19], pulse width [20,21], and repetition frequency on CCD damage [22,23]. Given the circumstances of nanosecond laser irradiation with multiple pulses, the experimental difficulty lies in capturing microscale changes within the nanosecond scale during a single-pulse cycle, owing to the nanosecond pulse width and millisecond pulse period. Hence, conducting finite element simulation analysis of nanosecond pulse laser-induced thermal damage on CCDs can effectively model temperature field variations within the CCD detector over multiple pulse cycles, thereby decreasing experimental expenses and improving research productivity.

This paper presents a finite element simulation study on the damage caused to a typical CCD by a multi-pulse nanosecond laser with a 532 nm wavelength. The study primarily focuses on the thermal damage mechanisms induced by ns-laser irradiation in detectors, where current experimental results also confirm that thermal effects dominate the damage progression [11]. Thus, non-thermal effects fall outside the scope of our work. Using the Fourier transient heat conduction equation [14], the study modeled the energy deposition, heat conduction, and heat diffusion process of the laser on the CCD’s multilayer structure. It analyzed the time evolution of the temperature field of the CCD detector under laser irradiation and explored the correlation between laser parameters, irradiation time, and CCD melting damage.

## 2. Model

### 2.1. Geometric and Physical Model

**Figure 1:**
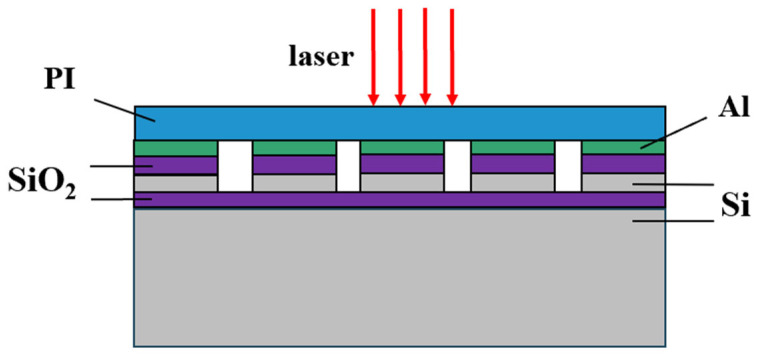
Multiple structure of the CCD.

**Figure 2:**
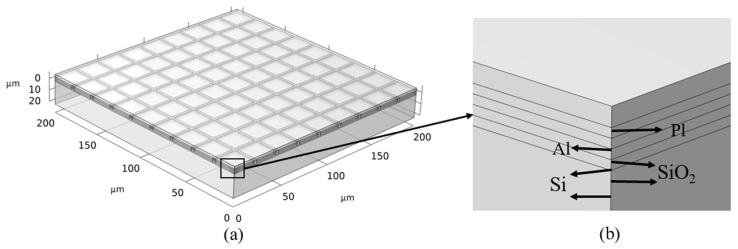
Geometric model: (**a**) three-dimensional model; (**b**) enlarged schematic of layered section.

Along the direction of laser incidence, the basic structure of a typical CCD detector consists of a microlens layer (PI), a light-shielding aluminum layer, a silicon dioxide layer, a polysilicon electrode, a silica insulating layer, and a silicon substrate [6], as shown in Figure 1. The geometric model is a 200 μm × 200 μm × 35 μm rectangle. The silicon substrate and silica insulating layer have thicknesses of 30 μm and 1 μm, respectively. Each polysilicon electrode, silicon dioxide layer, and light-shielding aluminum layer is 20 μm × 20 μm × 1 μm, arranged in a 9 × 9 array with a spacing of 2.5 μm on the upper surface of the silica insulating layer. The uppermost microlens layer has a thickness of 1 μm. The CCD multilayered structures are built up sequentially to form the final geometric model shown in Figure 2. The material properties of each layer are listed in Table 1.

When the nanosecond pulse laser irradiates the CCD, some of the laser beam passes through the microlens layer and the silica insulating layer because of their low absorption. The laser then reaches the photosensitive surface of the silicon substrate directly. Since silicon is a volume absorber, it acts as the volume heat source [6]. The laser heat source function can be described as follows:

(1) $$Q_{si,laser}=\alpha (1-R)e^{\left(-\alpha \cdot z\right)}f(t)\frac{2P_{0}}{\pi {\omega_{0}}^{2}}g(x,y)$$

Given the CCD’s opening rate (ratio of the effective photosensitive area to the total pixel area), laser irradiation also affects the light-shielding aluminum layer. The layer’s thickness exceeds the laser absorption depth in the model, resulting in the surface heat source being applied to the layer.

(2) $$Q_{Al,laser}=\beta f(t)\frac{2P_{0}}{\pi {\omega_{0}}^{2}}g(x,y)$$

where $\omega_{0}$ is the spot radius; *t* is the time; α is the absorption coefficient of the silicon for the laser (*α* = 7500 cm^−1^); *R* is the reflectivity of the photoreceptor region (the reflectivity *R* is 0.33 in the solid state and 0.72 in the liquid state); *β* is the absorption of the aluminum for the laser (*β* = 8%). The peak power *P*_0_ is as follows:

(3) $$P_{0}=\frac{E_{0}}{\tau }$$

where $E_{0}$ is the single-pulse energy and τ is the pulse width.

The temporal distribution function of the pulse laser beam can be expressed as follows:

(4) $$f(t)=\left\{\begin{array}{l} gp(t)\;,\;N/F\leq t\leq N/F+\tau \\ 0\;,\;N/F+\tau \leq t\leq \;(N+1)/F \end{array}\right.$$

where *F* is repetition frequency, *N* = 0, 1 2, ……; gp(t) is a Gaussian pulse function with a peak value of 1, and its standard deviation equals the pulse width.

(5) $$g(x,y)=e^{\left(-\frac{2\cdot (x^{2}+y^{2})}{\omega_{0}^{2}}\right)}$$

The function *g*(*x*, *y*) represents the spatial distribution of laser energy, which follows a Gaussian profile.

The temperature field during the deposition of laser energy on the CCD is controlled by the differential equation of heat conduction. Assuming uniform distribution of materials in each layer and isotropy, the temperature field control equation follows Fourier’s law of thermal conduction.

(6) $$\rho c\frac{\partial T}{\partial t}=k\left(\frac{\partial^{2}T}{\partial x^{2}}+\frac{\partial^{2}T}{\partial y^{2}}+\frac{\partial^{2}T}{\partial z^{2}}\right)+Q_{laser}$$

where $Q_{laser}$ is the laser heat source and ρ, c and k are the material density, specific heat, and heat conductivity, respectively.

Considering the radiation and convection heat loss on the surface of the material, the boundary conditions of the model can be expressed as follows:

(7) $$-k\frac{\partial T}{\partial n}=-\varepsilon \sigma ({T_{amb}}^{4}-{T_{w}}^{4})-h(T_{amb}-T_{w})$$

where $T_{w}$ is the detector surface temperature, $T_{amb}$ is the ambient temperature, *h* is the convective heat transfer coefficient, ε is the surface emissivity and σ is Stefan’s constant. The initial temperature of the model is set to 298 K.

### 2.2. Model Verification

**Figure 3:**
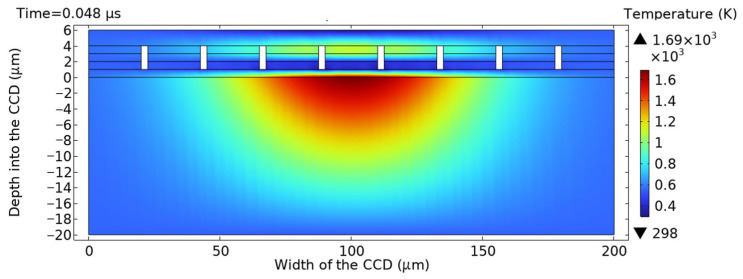
Cross-sectional temperature profile along the central axis at t = 0.048 μs.

**Figure 4:**
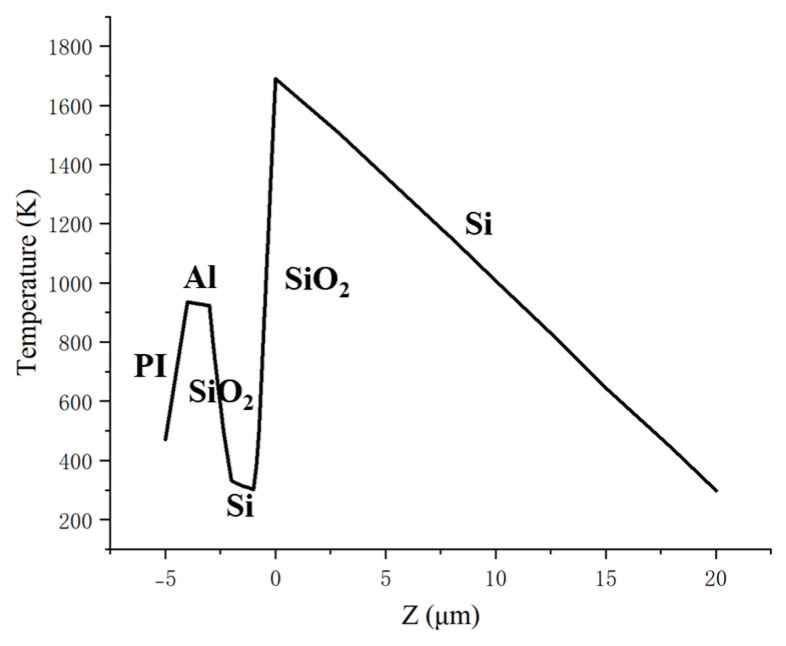
Temperature distribution along the laser irradiation direction at the beam center (t = 0.048 μs).

The energy density threshold of the nanosecond pulse thermally damaged CCD was simulated. The simulation parameters included a spot radius of 75 μm, pulse width of 10 ns, and single-pulse energy density of 0.76 J/cm^2^. The simulation results are depicted in Figure 3 and Figure 4, showing that the highest temperature of the CCD is on the upper surface of the silicon substrate when the time is 0.048 μs. The microlens layer, light-shielding aluminum layer, and silicon substrate have all reached their melting points, resulting in melting damage in the detector under the pulse irradiation. The simulation results showed that the melting damage occurred in the CCD detector when the energy density reached 0.76 J/cm^2^. This threshold value was consistent with experimental data from previous studies [20], validating the research method used in this paper.

## 3. Results and Discussion

### 3.1. Temporal Evolution of the CCD

**Figure 5:**
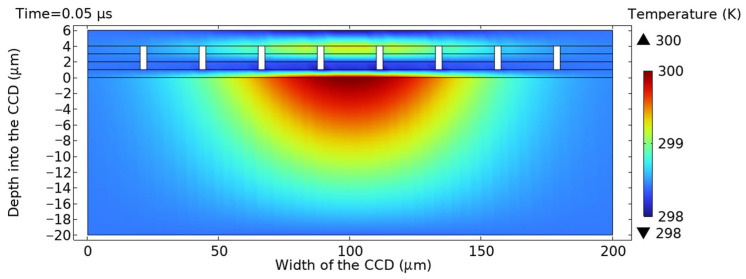
Cross-sectional temperature profile along the central axis at t = 0.05 μs.

The cumulative effect of temperature, when combined with multiple pulses, can lead to thermal damage to the CCD [6]. The simulation parameters for the laser heat source include a spot radius of 75 μm, repetition frequency of 10 kHz, pulse width of 100 ns and single-pulse energy of 0.05 mJ. As shown in Figure 5, when the initial laser pulse began irradiating the CCD target surface at t = 0.05μs, a slight temperature increase occurred in the central area, peaking at 300 K. No significant heat conduction was observed elsewhere.

**Figure 6:**
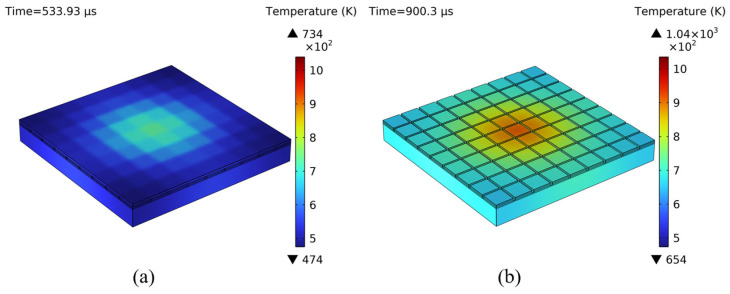
(**a**) Temperature field of the CCD at t = 533.93 μs. (**b**) Temperature field of the CCD at t = 900.3 μs.

After six pulses irradiated the CCD (t = 533.93 μs), the maximum temperature in the detector reached 741 K, as shown in Figure 6a. At this time, the microlens near the center of the irradiation point surpassed its thermal decomposition temperature of 710 K [17]. Consequently, the melting point of the microlens has been exceeded, which could significantly reduce the effectiveness of the microlens. As more laser pulses hit the CCD, the melting region of the microlens expanded, and the light-shielding aluminum layer absorbed and stored laser heat. As shown in Figure 6b, when the tenth laser pulse hit the CCD (t = 900.3 μs), the maximum temperature in the detector was 1040 K, and part of the light-shielding aluminum layer exceeded the melting point.

**Figure 7:**
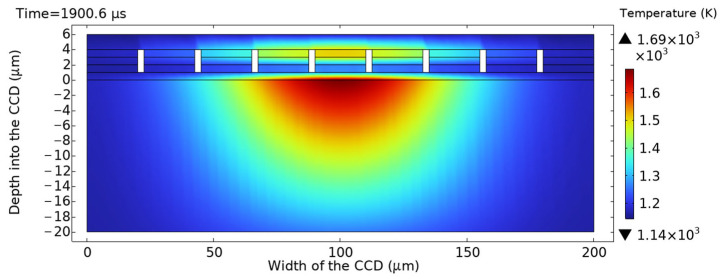
Cross-sectional temperature profile along the central axis at t = 1900.6 μs.

**Figure 8:**
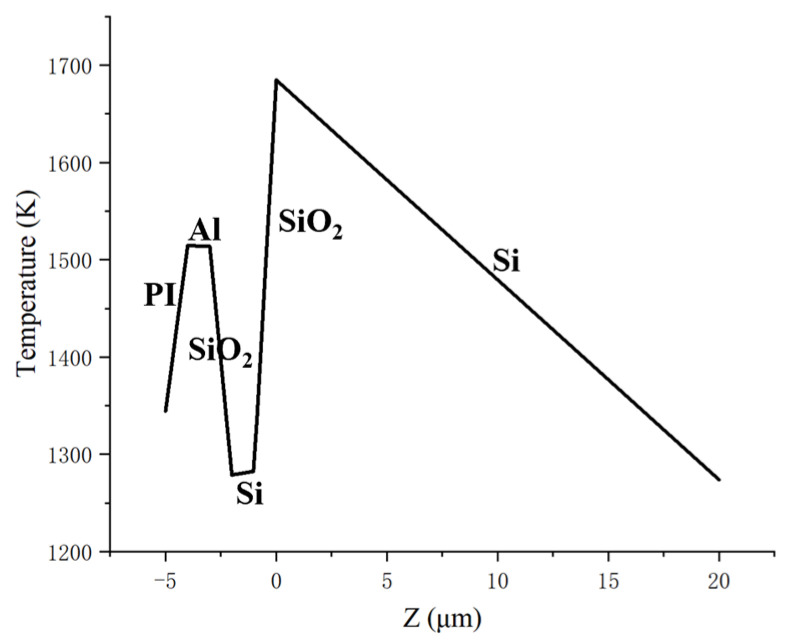
Temperature distribution along the laser irradiation direction at the beam center (t = 1900.6 μs).

As shown in Figure 7 and Figure 8, the twentieth laser pulse hit the CCD at t = 1900.6 μs, with the highest temperature of 1690 K on the upper surface of the silicon substrate. By this point, the melting point of the silicon substrate has been breached.

**Figure 9:**
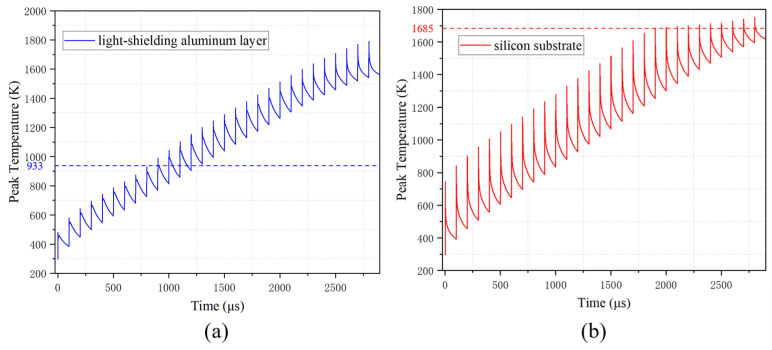
(**a**) Peak temperature development of the light-shielding aluminum layer over time (the blue dashed line indicates the melting point of aluminum). (**b**) Peak temperature development of the silicon substrate over time (the red dashed line indicates the melting point of silicon).

The peak temperature variation and melting damage size of the silicon substrate and light-shielding aluminum layer were analyzed to study the thermal accumulation effect over time. As shown in Figure 9, the heat from each pulse began to deposit on the surface before the previous pulse’s heat was completely dissipated, leading to a cumulative effect on temperature. The temperatures of both the aluminum layer and silicon substrate increased in a jagged manner with time as multiple laser pulses were applied. The aluminum layer reached its melting point after the tenth pulse, while the silicon substrate melted after the twentieth pulse. Notably, the temperature rise effect for the silicon substrate becomes slower from the twentieth pulse due to the increased laser reflectivity *R*.

**Figure 10:**
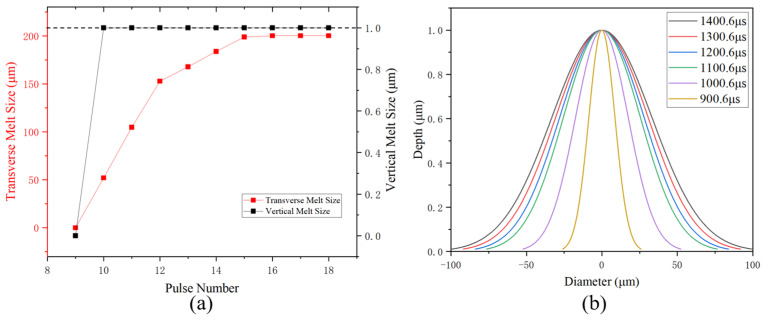
(**a**) Transverse melt size and vertical melt size with pulse numbers in light-shielding aluminum layer (the dark dashed line indicates the thickness of the aluminum layer). (**b**) Melt region shape in light-shielding aluminum layer at different times.

As shown in Figure 10, the light-shielding aluminum layer began to melt after the tenth laser pulse, forming a Gaussian distribution in the melt-damaged region. At this time, the maximum melting damage size in the transverse direction was about 51.90 μm, and in the vertical direction, it was about 1 μm, which was the thickness of the aluminum layer. With each pulse, the transverse size of the melt region gradually increases. By the twelfth pulse, the maximum transverse melting damage size reached 152.63 μm, extending beyond the spot diameter. Eventually, the temperature in the entire aluminum layer exceeds the melting point. It is noteworthy that lateral melt expansion per pulse becomes severely constrained after the twelfth pulse (at 1100.6 μs). This occurs because before the twelfth laser pulse, the melted area was narrower than the 150 μm laser spot. The laser directly heated this zone, causing it to melt rapidly. After this point, the melt became wider than 150 μm. However, areas outside the laser spot could only be heated by slow heat spread from the center. This slowed down the melting dramatically.

**Figure 11:**
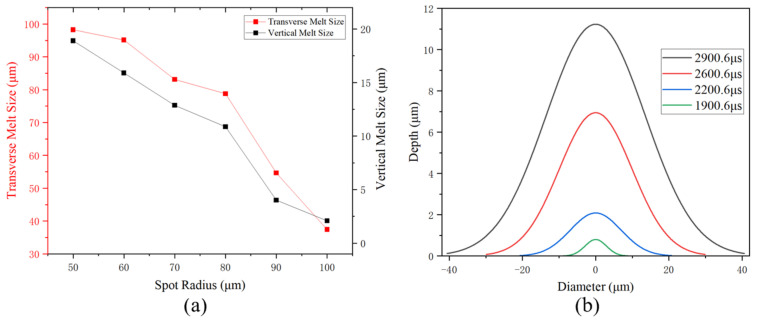
(**a**) Transverse melt size and vertical melt size with pulse numbers in silicon substrate; (**b**) melt region shape in silicon substrate at different times.

In Figure 11, the melting temperature of the silicon substrate was exceeded after the twentieth laser pulse. The melting damage was about 17.95 μm in the transverse direction and 0.80 μm in the vertical direction. The damage shape followed a Gaussian distribution, expanding in both directions with repeated laser pulse irradiations.

### 3.2. Influence of Laser Parameters on Thermal Damage

#### 3.2.1. Spot Radius

**Figure 12:**
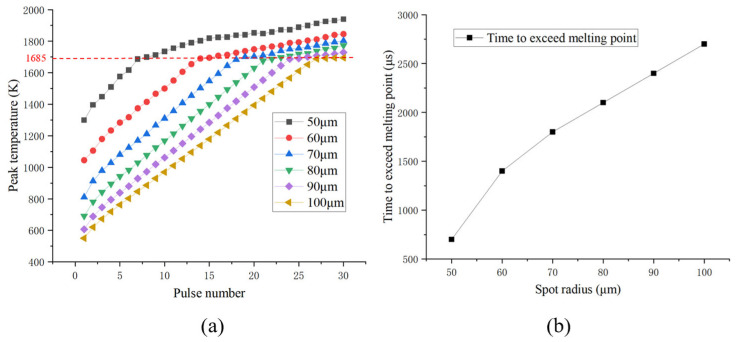
(**a**) Peak temperature of the silicon substrate irradiated by the laser with different spot radii, (**b**) melting time of the silicon substrate versus radius (the red dashed line indicates the melting point of silicon).

The size of the melt on the material surface is directly influenced by the deposition of laser energy. Different spot radii of the laser pulse will result in different spot shapes on the target, impacting the energy density distribution of the laser. Peak temperature variation of the silicon substrate irradiated by the laser with various spot radii is shown in Figure 12. Simulation parameters include a pulse energy of 0.05 mJ, repetition frequency of 10 kHz, pulse width of 100 ns, and laser spot radii ranging from 50 μm to 100 μm. These spot radii correspond to average power densities ranging from 6366.20 W/cm^2^ to 1591.55 W/cm^2^. The number of pulses required to induce thermal damage to the silicon substrate increased with a larger spot radius. The power density deposited within the spot is reduced, leading to a decrease in the amount of energy absorbed per unit area. This weakens the temperature rise effect of the pulse laser, eventually increasing the time of melting damage.

**Figure 13:**
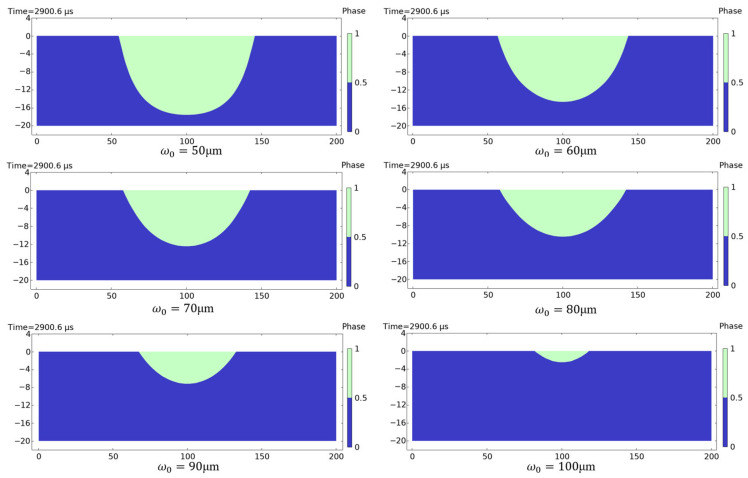
Cross-sectional melt morphology in silicon substrate after 30 pulses of irradiation (0 and 1 in the phase transition image denote solid phase and liquid phase, respectively).

**Figure 14:**
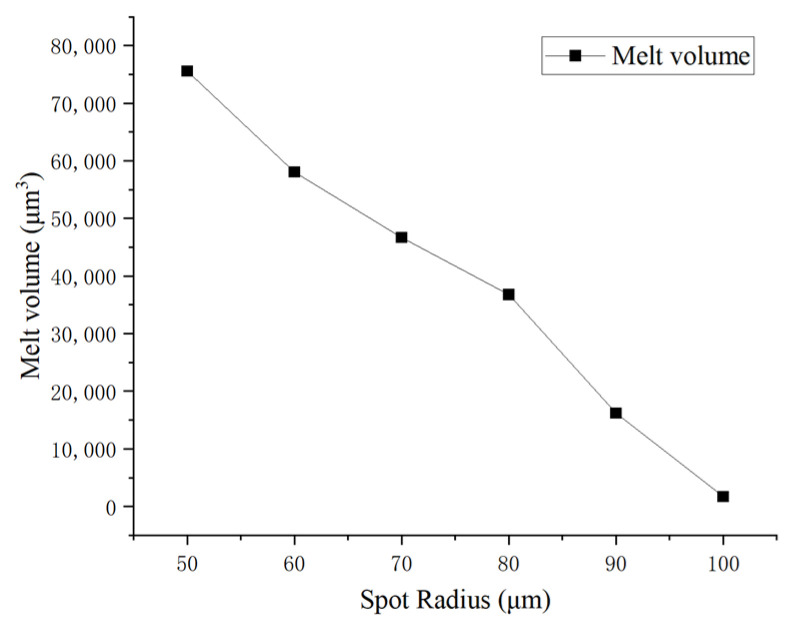
Melt volume vs. spot radius.

In Figure 13, the simulation shows the cross-sectional melt morphology in silicon substrate after 30 pulses of irradiation. The green areas indicated where melting occurred, while the blue areas remained unmelted. Figure 14 quantifies the progressive reduction in silicon melt volume (μm^3^) with increasing spot radius under 30-pulse irradiation. The laser with a radius of 50 μm, having the highest single-pulse power density, caused melting as early as the seventh pulse, resulting in the largest melt volume.

#### 3.2.2. Repetition Frequency and Pulse Width

The energy from the laser pulse is absorbed by the target surface and is gradually dissipated over time through heat conduction, thermal convection, and radiation. If the energy from one pulse is not fully dissipated before the next pulse is applied, the temperature of the surface will increase, leading to cumulative temperature effects. This effect becomes more pronounced with each subsequent pulse, potentially causing melting damage to the material. The time between pulses is determined by the laser irradiation time and pulse period, with the pulse width and repetition frequency being the main factors affecting the cumulative temperature effect.

1. Repetition frequency

**Figure 15:**
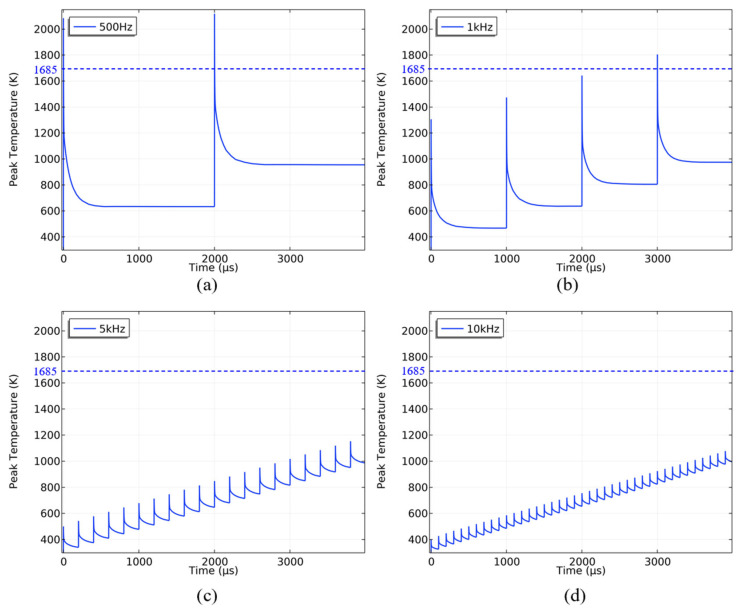
The variation in the silicon substrate temperature when the repetition frequency is (**a**) 500 HZ, (**b**) 1 kHZ, (**c**) 5 kHZ, and (**d**) 10 kHZ, respectively (the blue dashed line indicates the melting point of silicon).

The frequencies tested were 500 Hz, 1 kHz, 5 kHz, and 10 kHz. The laser output maintained an average power density of 636.62 W/cm^2^ for 4000 μs, with a spot radius of 100 μm and a pulse width of 100 ns. The silicon substrate temperature variation over time is shown in Figure 15.

When a silicon substrate was irradiated by a laser with a repetition frequency of 500 Hz, the temperature reached 2088 K after the first pulse and dropped to 633 K before the second pulse, resulting in a temperature difference of 1455 K. As the repetition frequency increased, the temperature difference between pulses gradually decreased. With a laser repetition frequency of 10 kHz, the temperature difference between each pulse was about 75 K. A laser with a lower repetition frequency can cause melting damage to the silicon substrate due to higher peak power density, while a higher repetition frequency may not melt the substrate within a certain time period.

2. Pulse width

**Figure 16:**
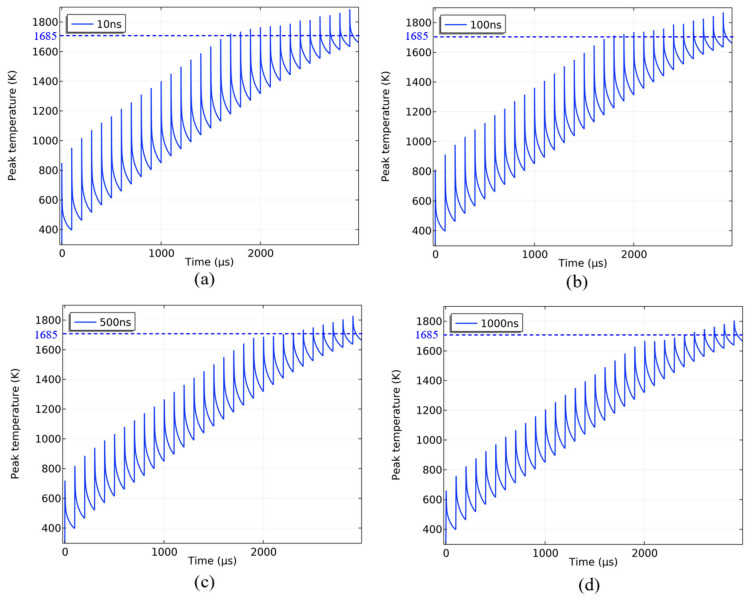
The variation in the silicon substrate temperature when the pulse width is (**a**) 10 ns, (**b**) 100 ns, (**c**) 500 ns, and (**d**) 1000 ns, respectively (the blue dashed line indicates the melting point of silicon).

The simulation investigated the impact of varying pulse widths (10 ns, 100 ns, 500 ns, and 1000 ns) on CCD thermal damage. Thirty pulse cycles were simulated with an average power density of 3248.06 W/cm^2^, a repetition frequency of 10 kHz, and a spot radius of 70 μm. The peak temperature variation profile of the silicon substrate is shown in Figure 16.

The temperature rise of the silicon substrate followed a dynamic process, where it rapidly increased to the melting point with the rise of pulse number and then increased slowly. Changing the pulse width could not significantly alter this situation, and the time of melting damage caused by different pulse widths remained basically the same. The peak temperature of the silicon substrate was most pronounced at a pulse width of 10 ns, reaching 847 K at the first pulse. However, the temperature dropped back to around 400 K before the second pulse. As the pulse width increased from 10 ns to 100 ns, the heat dissipation between pulses decreased resulting in a smaller temperature difference before and after the silicon substrate melted. Short pulse width lasers with higher peak power will have a more significant effect on pulse-induced temperature rise, but the accumulative effect of temperature for long pulse width lasers compensated for this difference. Therefore, in nanosecond pulse width, when the repetition frequency and average power density are constant, the thermal damage effect on the CCD between different pulse widths is not significant.

## 4. Conclusions

The finite element method was used to simulate CCD thermal damage caused by a multi-pulse nanosecond laser. The simulation results indicated that the thermal damage mechanism of the CCD evolved gradually due to thermal accumulation between pulses. Damage first occurred in the microlens layer, followed by significant effects on the light-shielding aluminum with increasing pulse numbers. The aluminum melted under the thermal effect of the laser. Then, the silicon substrate started to suffer from melting damage.

When the spot radius of the laser changed, the irradiated area on the target surface and the pulse energy density also changed. A smaller spot radius increased the temperature rise effect of a single pulse, leading to quicker melting of the silicon substrate. Reducing the repetition frequency extended the cooling time between laser pulses, weakening the thermal accumulation effect on the CCD. However, maintaining the average laser power while decreasing the repetition frequency increased the peak power density of each pulse, causing quicker material damage. Shorter pulse widths resulted in higher peak power densities, but also increased heat dissipation. This compensated for the difference in temperature rise between long and short pulse lasers. Therefore, changing pulse width in the nanosecond range cannot significantly affect thermal damage to the CCD.

## Figures and Tables

**Table 1:** The thermodynamic parameters of materials.

Parameters	PI	Al	SiO_2_	Si
Density/(kg/m^3^)	1530	2709	2640	2330
Thermal conductivity/(W/m·K)	0.12	254	1.3	27
Specific heat/(J/kg·K)	1090	1050	787	1009
Young modulus/(Pa)	4 × 10^9^	7 × 10^10^	7.78 × 10^10^	1.3 × 10^11^
Thermal expansion coefficient/(K^−1^)	2.0 × 10^−5^	2.29 × 10^−5^	5.0 × 10^−5^	2.0 × 10^−5^
Poisson ratio	0.3	0.3	0.17	0.2
Melting point/K	710	932	1880	1685

## Data Availability

The data presented in this study are available on request from the corresponding author. Informed consent was obtained from all subjects involved in the study.
